# Supplementary material for: A yeast phenomic model for the influence of Warburg metabolism on genetic buffering of doxorubicin
Source: Cancer Metab. 2019 Oct 23;7:9. doi: 10.1186/s40170-019-0201-3 (PMC6806529; doi:10.1186/s40170-019-0201-3)
Supplement: Supplementary file 9 — Additional file 9. GO term-specific heatmaps for REMc/GTF-enriched clusters. GO term-specific heatmaps for significant GO process terms were generated as described in methods and Figs. 3 and 4. Any related child terms are presented in subsequent pages of the parent file name. GO terms with more than 100 children, with 2 or fewer genes annotated to the term, or a file size over 300KB are not shown. All heatmaps are generated with the same layout (see Figs. 3 and 4). [file 40170_2019_201_MOESM9_ESM.bz2 › Additional_File9_GOTermHeatmaps/Additional_File9_GOTermHeatmap/transcription_elongation_from_RNA_polymerase_II_promoter.pdf]

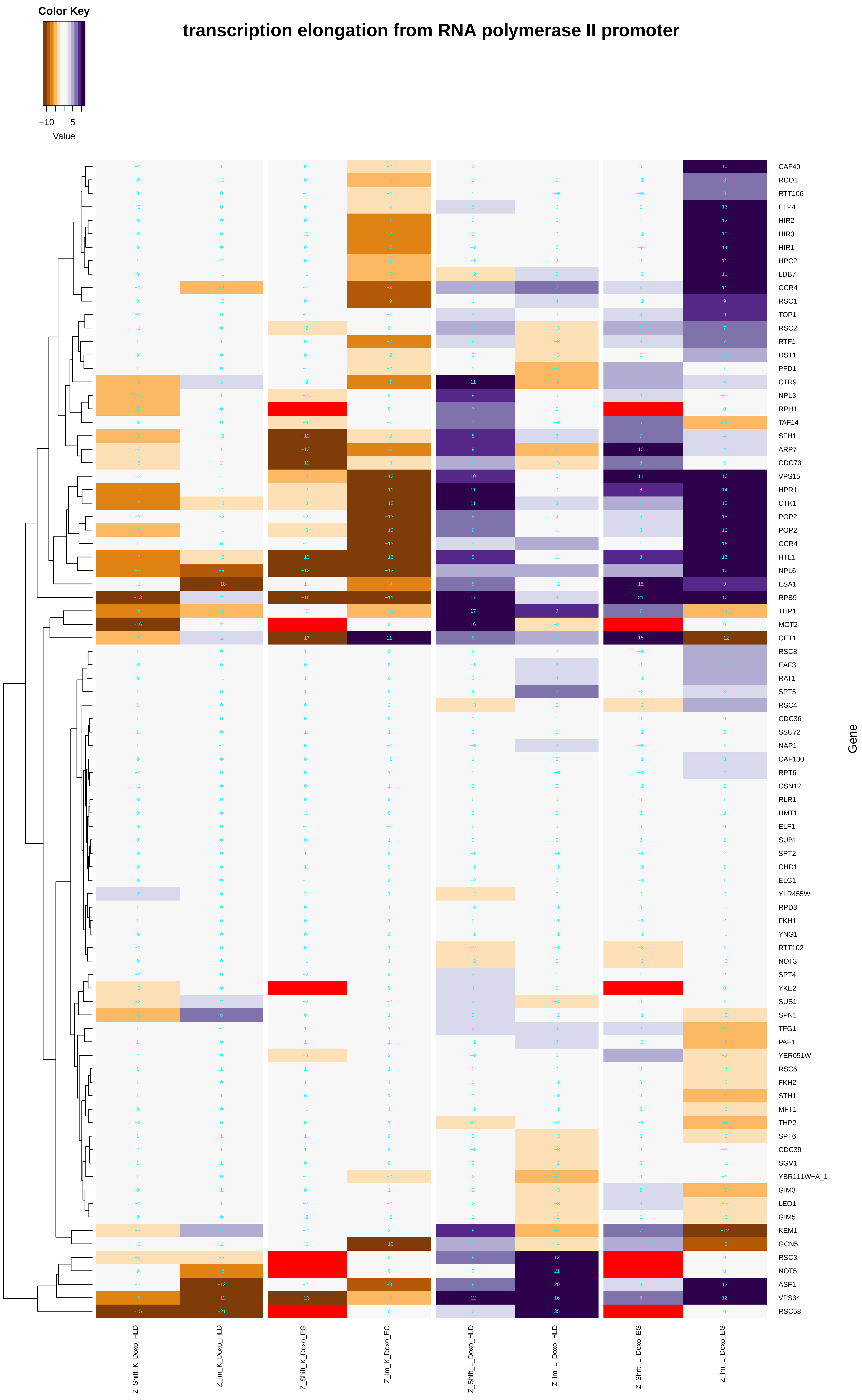
transcription elongation from RNA polymerase II promoter
Color Key
−10
5
Value
Gene
CAF40
RCO1
RTT106
ELP4
HIR2
HIR3
HIR1
HPC2
LDB7
CCR4
RSC1
TOP1
RSC2
RTF1
DST1
PFD1
CTR9
NPL3
RPH1
TAF14
SFH1
ARP7
CDC73
VPS15
HPR1
CTK1
POP2
POP2
CCR4
HTL1
NPL6
ESA1
RPB9
THP1
MOT2
CET1
RSC8
EAF3
RAT1
SPT5
RSC4
CDC36
SSU72
NAP1
CAF130
RPT6
CSN12
RLR1
HMT1
ELF1
SUB1
SPT2
CHD1
ELC1
YLR455W
RPD3
FKH1
YNG1
RTT102
NOT3
SPT4
YKE2
SUS1
SPN1
TFG1
PAF1
YER051W
RSC6
FKH2
STH1
MFT1
THP2
SPT6
CDC39
SGV1
YBR111W−A_1
GIM3
LEO1
GIM5
KEM1
GCN5
RSC3
NOT5
ASF1
VPS34
RSC58
Z_Shift_K_Doxo_HLD
Z_Im_K_Doxo_HLD
Z_Shift_K_Doxo_EG
Z_Im_K_Doxo_EG
Z_Shift_L_Doxo_HLD
Z_Im_L_Doxo_HLD
Z_Shift_L_Doxo_EG
Z_Im_L_Doxo_EG
